# Anionic Polymer Brushes for Biomimetic Calcium Phosphate Mineralization—A Surface with Application Potential in Biomaterials

**DOI:** 10.3390/polym10101165

**Published:** 2018-10-18

**Authors:** Tobias Mai, Karol Wolski, Agnieszka Puciul-Malinowska, Alexey Kopyshev, Ralph Gräf, Michael Bruns, Szczepan Zapotoczny, Andreas Taubert

**Affiliations:** 1 Institute of Chemistry, University of Potsdam, D-14476 Potsdam, Germany; mai.tobias@gmx.net; 2 Faculty of Chemistry, Jagiellonian University, Gronostajowa 2, 30-387 Krakow, Poland; wolski@chemia.uj.edu.pl (K.W.); puciul@chemia.uj.edu.pl (A.P.-M.); 3 Institute of Physics and Astronomy, University of Potsdam, D-14476 Potsdam, Germany; kopyshev@uni-potsdam.de; 4 Institute of Biochemistry and Biology, University of Potsdam, D-14476 Potsdam, Germany; rgraef@uni-potsdam.de; 5 Institute for Applied Materials and Karlsruhe Nano Micro Facility (KNMF), Karlsruhe Institute of Technology, D-76344 Eggenstein-Leopoldshafen, Germany; michael.bruns@kit.edu

**Keywords:** polymer brushes, calcium phosphate, hydroxyapatite, carbonated apatite, bone mimic, biocompatibility, *Dictyostelium discoideum*

## Abstract

This article describes the synthesis of anionic polymer brushes and their mineralization with calcium phosphate. The brushes are based on poly(3-sulfopropyl methacrylate potassium salt) providing a highly charged polymer brush surface. Homogeneous brushes with reproducible thicknesses are obtained via surface-initiated atom transfer radical polymerization. Mineralization with doubly concentrated simulated body fluid yields polymer/inorganic hybrid films containing AB-Type carbonated hydroxyapatite (CHAP), a material resembling the inorganic component of bone. Moreover, growth experiments using *Dictyostelium discoideum* amoebae demonstrate that the mineral-free and the mineral-containing polymer brushes have a good biocompatibility suggesting their use as biocompatible surfaces in implantology or related fields.

## 1. Introduction

The expected lifetime at birth has dramatically increased over the last 150 years. For example, life expectancy in Germany has more than doubled—from 37 years in 1871 to about 80 years in 2010 [1]. Among others, this is due to improved nutrition supplies and society-induced changes to physical activity patterns. However, as a consequence of this lifetime extension, diseases that were virtually unknown 200 years ago have become major factors in today’s health industries. These diseases include osteoporosis, chondrocalcinosis, kidney stones, atherosclerosis, but also caries and calculus.

Many of the diseases mentioned above are associated with the (unwanted or uncontrolled) deposition or dissolution of, mostly calcium-based, mineral deposits in the body. Biological mineral deposition is a highly complex physico-chemical process that is among the key processes to control in biomaterials design. Often, (biological) mineral formation and dissolution occur at an interface. This has triggered a number of studies on the effects of surfaces and interfaces on mineral deposition, notably calcium phosphate (CP) [2,3,4,5,6,7,8].

Understanding and controlling surface- and interface-controlled mineral formation may also be helpful for improving the design of biomaterial surfaces, because initially, the incorporation of an implant is again controlled by the interaction of the implant surface with the body. As a result, there is a need for tailor-made (model) surfaces that enable (i) the investigation of mineral formation and dissolution; (ii) the behavior of these surfaces in vitro and (iii) in vivo. Polymer brushes are one strategy for investigating these phenomena and processes [9,10,11,12,13,14].

The current work focuses on negatively charged polymer brushes grafted on silicon wafers as a model surface. The 3-sulfopropyl methacrylate (SPM) chemistry employed here is inspired by the fact that block copolymers of poly(ethylene oxide) (PEO) and poly(3-sulfopropyl methacrylate) (PSPM) exhibit very strong effects on CP mineralization [15]. Importantly, PSPM chains are highly negatively charged over a much broader pH range than previous examples of polymer surfaces [5]. Indeed, several studies [10,12,16] show that surfaces carrying a large number of sulfonate groups lead to metal ion enrichment and subsequent precipitation of a variety of minerals.

Somewhat surprisingly, however, there is only one study on CP mineralization demonstrating favorable effects for calcium phosphate mineralization both with negatively and positively charged weak polyelectrolyte surfaces [5]. The current study demonstrates that strong polyelectrolyte brushes may be even more attractive for the generation of hybrid thin layers than the brushes studied so far because they are highly charged from ca. pH 2 up; this makes them very interesting for the generation of surfaces that remain charged under physiological conditions, for example on an implant surface. The surfaces described here are thus much better models (and potential surface modifiers for implants) than our previous examples [5] for the investigation of bioinspired surface mineralization.

## 2. Materials and Methods

Sulfuric acid (98% for analysis), aqueous hydrogen peroxide (30% for synthesis), benzene (ACS, ISO, Reag. Ph. Eur. for analysis), dichloromethane (DCM; ACS, ISO, Reag. Ph. Eur. for analysis), ethanol (>96% not denaturized), and chloroform (ACS, ISO, Reag. Ph. Eur. for analysis) were purchased from VWR international^®^ (Darmstadt, Germany) and 3-iodopropyltrimethoxysilane, 3-aminopropyltrimethoxysilane (97%) and (*N*-methylaminopropyl)trimethoxysilane from abcr^®^ (Karlsruhe, Germany). Poly(ethylene oxide) (PEO4600, nominal M_W_ = 4600 g/mol), *N*,*N*,*N*′,*N*′-tetramethylethylenediamine (TMEDA) (ReagentPlus^®^, 99%, freshly distilled), 3-sulfopropylmethacrylate potassium salt (SPM) (98%), triethylamine (≥99%) and 2M lithium diisopropylamine (LDA) in tetrahydrofurane (THF), *N*-propyl gallate and α-bromoisobutyryl bromide (98%) were purchased from Sigma Aldrich^®^ (Taufkirchen, Germany). HL5c medium was obtained from Formedium (Hunsanton, UK), 24 well cell culture plates from Sarstedt (Nümbrecht, Germany), glutaraldehyde (0.5%) from Plano (Wetzlar, Germany), and the silicon wafer with diameter of 150 mm and <100> orientation from SI-MAT (Kaufering, Germany). All chemicals were used as received.

Ultrapure water with a resistivity of 18.2 MΩ∙cm was obtained fresh from an ELGA Pure Lab ultra machine (Celle, Germany). Dry solvents were prepared according to previously published procedures [17].

### 2.1. Preparation

Prior to further use, wafer sections of ~1 × 1 cm² were generously rinsed with ethanol, chloroform, ethanol, and ultrapure water. After drying with an Ar-stream the wafers were treated with fresh piranha solution (1:1 (*v*/*v*) sulfuric acid/aqueous hydrogen peroxide) for 30 s, again generously rinsed with ultrapure water and finally dried with argon.

### 2.2. Sample Nomenclature

**Figure 1:**
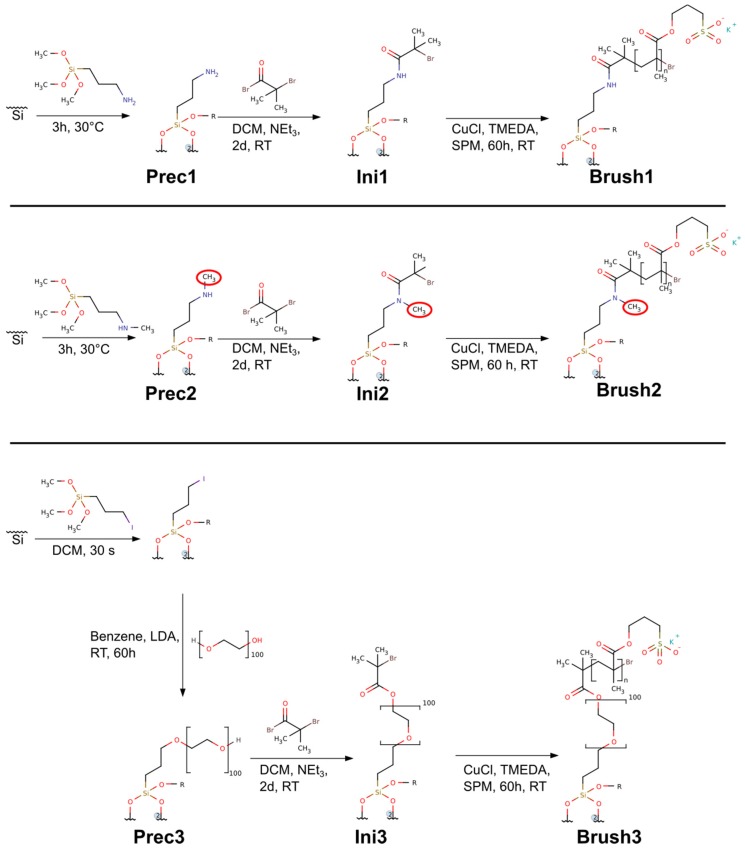
Synthetic route towards the brushes used in this study.

The sample numbers are kept the same throughout the text: for example, the precursor Prec1 is transformed into the initiator Ini1 which is then transformed into the polymer brush Brush1 and finally into the mineralized sample Min1 (Figure 1). The same applies to the other samples.

### 2.3. Precursors—Prec1, Prec2, Prec3

The precursors Prec1 and Prec2 were grafted to the silicon surfaces using the same strategy for all samples. At the bottom of a 15 mL centrifuge tube 30 µL of 3-aminopropyltrimethoxysilane (Prec1) or *N*-methylaminopropyl trimethoxysilane (Prec2) were deposited. Then, the activated wafers (after the piranha solution treatment) were exposed to the precursor vapor by placing them perpendicularly on top of the precursor in the centrifuge tube without touching the liquid. After 3 h at 30 °C the wafers were removed and rinsed with copious amounts of ethanol, chloroform, ethanol, ultrapure water, and finally dried in a stream of argon. If not used immediately, the wafers were stored in deionized water.

Prec3 was obtained by covering a freshly prepared wafer with 3-iodopropyl trimethoxysilane for 2 min followed by generously rinsing the wafer with ethanol, chloroform, ethanol, and ultrapure water before drying in a stream of argon. If not used immediately, the wafer was stored in deionized water. Subsequently, the dry wafers were inserted into 15 mL centrifuge tubes filled with dry benzene to completely cover the wafers. After the addition of 100 mg of PEO4600, the tube was closed and shaken for 2 min to dissolve the polymer. Then 100 µL of 2 M LDA solution in THF was added and the closed tube was shaken for 5 s and allowed to stand for 60 h. The wafers were removed from the now brown liquid and generously rinsed with ethanol, chloroform, ethanol, and water and finally dried in a stream of argon.

### 2.4. ATRP Initiators—Ini1, Ini2, Ini3

The ATRP initiators Ini1, Ini2 and Ini3 were obtained using an identical procedure. A wafer covered with a monolayer of a given precursor (Prec1, Prec2, or Prec3) was immersed in 3 mL of dry DCM or chloroform in a 15 mL centrifuge tube. Then 139 µL of triethylamine and 123 µL of α-bromoisobutyryl bromide were added. The tube was closed, shaken, and left for 60 h for reaction completion at room temperature. After removal from the liquid, the wafer was generously rinsed with ethanol, chloroform, ethanol, and ultrapure water. If not used immediately the wafer was stored in deionized water.

### 2.5. Brushes—Brush1, Brush2, Brush3

All polymer brushes Brush1, Brush2, Brush3 were synthesized with the same strategy using an established protocol [15,18]. A 5 mL screw lid vial was cleaned with concentrated nitric acid (69%) and ultrapure water just before use. Thereafter a dry, initiator-modified wafer (Ini1, Ini2, Ini3) was deposited in the clean vial and the vial was closed with a septum. Subsequently 1 g of SPM and 11 µL of TMEDA were dissolved in a second vial in 1 mL of ultrapure water and the solution was deoxygenated for 5 min with argon. Afterwards, the solution was transferred to a vial containing 10 mg of copper(I) chloride. This mixture was stirred vigorously until the copper(I) chloride was completely dissolved. This solution was then transferred to the first vial containing the wafer. After 12 h at room temperature the wafer was removed and generously rinsed with ultrapure water before immersing it into a vial of ultrapure water for an hour. Finally, the wafer was again rinsed with ultrapure water and dried in a stream of argon. If not used immediately, the samples were again stored in water. Figure 1 shows the route for brush synthesis.

### 2.6. Mineralization—Min1, Min2, Min3

Mineralization of the brushes was achieved using an identical approach for all samples Min1, Min2, and Min3 [15,18]. In brief, for mineralization, 15 mL of the Ca-containing component of doubly concentrated simulated body fluid (Ca-2SBF) and 15 mL of the phosphate component of doubly concentrated SBF (P-2SBF) were mixed and stirred. Then a wafer was placed at the center of the reaction vessel with the brush side facing downwards to avoid sedimentation of CP formed in solution onto the brush surfaces. After ca. 5 min, 2.5 mL of a 0.1 M CaCl_2_ solution were added without stirring to induce mineral formation. After 24 h, the wafer was removed from the solution and residual liquid on the wafer was removed with a stream of argon, but the wafer was not dried completely at this step. Afterwards, the wafer was rinsed generously with distilled water and dried in a stream of argon. The entire mineralization procedure was repeated once to ensure uniform mineralization.

### 2.7. Atomic Force Microscopy (AFM)

AFM experiments were done in air on a MultiMode (Bruker, Poznan, Poland) microscope working in tapping^®^ mode (silicon cantilevers with a nominal spring constant of 40 N m^−1^) and on a Dimension Icon (Bruker, Poznan, Poland) microscope working in the PeakForce QNM^®^-Mode (silicon cantilevers with a nominal spring constant of 0.4 N m^−1^). The dry thicknesses of the brushes were determined using the AFM height measurements at the edges of scratches formed in the brush layers using tweezers. For image analysis, processing, and presentation GWYDDION 2.34 [19] (http://gwyddion.net/) was used. Thickness determination on scratched surfaces [20,21] was repeated 9–10 times for Brush1 and Brush2. Brush3 samples were highly inhomogeneous and thickness measurements were not reproducible. All samples were rinsed with ultrapure water and dried with nitrogen before analysis.

### 2.8. Contact Angle Measurements

Contact angle measurements were done on a KSV-CAM 100 contact angle meter (KSV Instruments, Helsinki, Finland). Before analysis, the samples were rinsed with water and dried with nitrogen or argon.

### 2.9. Scanning Electron Microscopy (SEM)

SEM was done on a JEOL JSM-6510 (Freising, Germany) with a tungsten filament. All measurements were performed at 0.5–30 kV. All samples were carbon-coated prior to imaging using a POLARON CC7650 carbon coater. All samples were glued on an aluminum sample holder using a conductive glue-pad (Plano) and the sample and the sample holder were electrically contacted by copper tape to avoid or at least reduce sample charging because charging of such non-conductive samples is very common.

### 2.10. Infrared Reflection Absorption Spectroscopy (IRRAS)

IRRAS data were obtained on a Nicolet iS10 spectrometer with grazing-angle reflectance accessory set at 84° for all measurements. Spectra were measured from 650 to 4000 cm^−1^ using 512 scans for averaging and a resolution of 8 cm^−1^. Before analysis, all samples were rinsed with water and dried with argon.

### 2.11. X-Ray Photoelectron Spectroscopy (XPS)

XPS measurements were performed using a K-Alpha+ instrument (ThermoFisher Scientific, East Grinstead, UK). Data acquisition and processing using the Thermo Avantage software is described elsewhere [22]. All samples were analyzed using a micro-focused, monochromated Al Kα X-ray source (400 μm spot size). The K-Alpha charge compensation system was employed during analysis, using electrons of 8 eV energy and low-energy argon ions to prevent any localized charge build-up. The spectra were fitted with one or more Voigt profiles (binding energy uncertainty: ±0.2 eV). The analyzer transmission function, Scofield sensitivity factors [23], and effective attenuation lengths (EALs) for photoelectrons were applied for quantification. EALs were calculated using the standard TPP-2M formalism [24].

All spectra were referenced to the C1s peak of hydrocarbon at 285.0 eV binding energy controlled by means of the well-known photoelectron peaks of metallic Cu, Ag, and Au. Sputter depth profiles were performed using a raster scanned Ar+ ion beam at 1.0 keV and 30° angle of incidence.

### 2.12. Cell Culture Experiments

*Dictyostelium discoideum* cells expressing the green fluorescent GFP-LimΔcoil fusion protein [25] were cultivated on mineralized and unmineralized wafers at 21 °C in HL5c medium (Formedium, Hunsanton, UK). All samples were incubated for 60 h; then the adhering cells were fixed with glutaraldehyde (0.5%) [26]. Actin appears green as GFP-Limcoil fusion protein binds to F-actin. Microtubules were visualized in red using the monoclonal anti-α-tubulin antibody YL1/2 and the anti-rat-antibody AlexaFluor-568. Cell nuclei were labeled in blue with 4′,6-diamidino-2-phenylindol dihydrochloride (DAPI). *N*-propylgallate (2%) was used as anti-bleaching agent and samples were mounted in Mowiol [26]. Wide-field microscopy was done on a Zeiss CellObserver HS/Axiovert 200M system with a PlanApo 100×/1.4 N.A. Lens and an Axiocam MRm Rev. 3 CCD Camera. z-Stacks were recorded at a distance of 0.25 µm. Iterative deconvolution of microscopic images with a measured point spread function was performed with Zeiss Axiovision 4.8. Data analysis was carried out with ImageJ 1.48k (Rasband, W.S., ImageJ, U.S. National Institutes of Health, Bethesda, MD, USA, http://imagej.nih.gov/ij/, 1997–2014.)

### 2.13. IRRAS of Brush1, Brush2, Brush3, Min1, Min2, Min3

Brush1. IRRAS: 2960 cm^−1^, C–H asymmetric stretching vibration; 2897 cm^−1^, C–H symmetric stretching vibration; 1725 cm^−1^, C=O stretching vibration of saturated ester; 1448 cm^−1^, C–H asymmetric deformation of CH_3_; 1475 cm^−1^ shoulder, C–H symmetric deformation of CH_3_; 1262 cm^−1^, symmetrical Si–C bending [27,28,29] 1190 cm^−1^, symmetric stretching vibration of SO_3_; 1107 cm^−1^ Si–O–Si stretching [27,28,29]; 1048 cm^−1^, asymmetric stretching vibration of SO_3_. Molecular weights (M_W_) of the individual polymer chains could not be determined; for M_W_ determination the polymers would need to be removed from the surfaces; this is particularly difficult with the rather heterogeneous Brush3, but is problematic for all samples.

Brush2. IRRAS: 2960 cm^−1^, C–H asymmetric stretching vibration; 2897 cm^−1^, C–H symmetric stretching vibration; 1725 cm^−1^, C=O stretching vibration of saturated ester; 1448 cm^−1^, C–H asymmetric deformation of CH_3_; 1475 cm^−1^, C–H symmetric deformation of CH_3_; 1246 cm^−1^, symmetrical Si–C bending [27,28,29]; 1215 cm^−1^, symmetric stretching vibration of SO_3_; 1107 cm^−1^ Si–O–Si stretching [27,28]; 1048 cm^−1^, asymmetric stretching vibration of SO_3_.

Brush3. IRRAS: 2960 cm^−1^, C–H asymmetric stretching vibration; 2897 cm^−1^, C–H symmetric stretching vibration; 1725 cm^−1^, C=O stretching vibration of saturated ester; 1448 cm^−1^, C–H asymmetric deformation of CH_3_; 1263 cm^−1^, symmetrical Si–C bending [27,28,29]; 1190 cm^−1^, symmetric stretching vibration of SO_3_; 1107 cm^−1^ Si–O–Si stretching [27,28]; 1048 cm^−1^, asymmetric stretching vibration of SO_3_.

Min1. IRRAS: 1726 cm^−1^,C=O vibration; 1107 cm^−1^ ν_3_-PO_4_^3−^ vibration of phosphates [30,31,32] 1445 cm^−1^ ν_3_ vibrations of CO_3_^2−^ [30,31,32] 893 cm^−1^ ν_2_ vibrations of CO_3_^2−^ [30,31].

Min2. IRRAS: 1726 cm^−1^, C=O vibration; 1109 cm^−1^, PO_4_^3−^ ν_3_ vibration of phosphates [30,31,32]; 1416 cm^−1^, ν_3_ of CO_3_^2−^ [30,31,32]; 903 cm^−1^, A-type CO_3_^2−^ substitution of apatites [30,31]; 871 cm^−1^, B-type CO_3_^2−^ substitution of apatites [30,31].

Min3. IRRAS: 1490 cm^−1^ ν_3_ of carbonate [30,31]; 1043 cm^−1^ ν_3_-PO_4_^3−^ vibration of phosphates [30,31,32]; 862 cm^−1^ ν_2_ of CO_3_^2−^ [30,31].

## 3. Results

### 3.1. Polymer Brushes

Contact angle (CA) measurements were used to assess the surface modifications (Table 1). CA measurements provide qualitative insight into the surface modification. Prec1 and Prec2 have CA values of around 20° indicating a rather hydrophilic surface. After modification to Ini1 or Ini2, the surfaces become more hydrophobic with CAs values of ca. 75°. Finally, after polymerization, the CA is again lower (ca. 7°), proving that the polymerization reaction leads to a hydrophilic surface.

Prec3 is slightly different in that the CA is ca. 15°, which is slightly lower than the 20° found for Prec1 and Prec2. Consistent with this observation, the CA of Ini3 is ca. 50°. This is again lower than what is observed for Ini1 and Ini2. After polymerization of the anionic brush, however, the CA is also 7°, identical to the Brush1 and Brush2 surfaces.

Brush thickness was determined via AFM on scratched surfaces; thicknesses are given in Table 1. Brush1 and Brush2 exhibit similar thicknesses (ca. 80–90 nm) while the largest variation is observed for Brush3.

The somewhat different behavior of Brush3 compared to the other brushes is further confirmed when the efficiency of the surface modification is considered. All polymerization reactions work, but various samples of Brush3 often exhibit (on the same wafer) individual regions that contain the polymer brush while other regions on the same wafer are not (fully) covered with the brush. Overall, only ca. 50% of the Brush3 wafers afford optically faultless and homogeneously covered wafer surfaces. It is likely due to non-uniform attachment of PEO to the surface producing an inhomogenous distribution of the subsequently attached ATRP initiator on the surface. To exclude artifacts in the data by non-uniform surfaces, only completely covered and homogeneous samples where used for the subsequent experiments.

**Figure 2:**
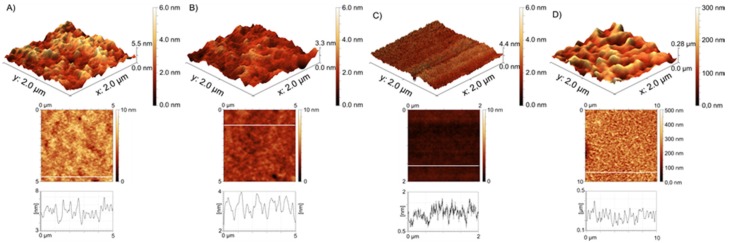
3D AFM topographic views (top row), height images (middle row), and example height profiles (bottom) of (**A**) Brush1 (R_a_ = 0.3 nm; R_z_ = 1.64 nm); (**B**) Brush2 (R_a_ = 0.12 nm; R_z_ = 0.61 nm); (**C**) Brush3 with a thickness of 30 nm (R_a_ = 0.12 nm; R_z_ = 0.69 nm); and (**D**) Brush3 with a thickness of 300 nm (R_a_ = 26 nm; R_z_ = 154 nm). A detailed analysis can be found in the supporting information.

Figure 2 shows AFM images of all brushes used in this study. Brush1 and Brush2 are quite similar in terms of the overall appearance of the surface morphology and thickness (see above). This is indicated by similar roughness values (R_a_ = 0.3 nm for Brush1 and 0.12 nm for Brush2). AFM results, therefore, suggest that the difference between the starting groups—one additional methyl group in case of Brush2—does not significantly affect the overall polymerization reaction.

However, as stated above, Brush3 shows much less reproducible results in spite of identical conditions used for polymerization; Figure 2 therefore shows two different brushes of this type. One brush is very thin with 30 nm thickness and the other one is much thicker (ca. 300 nm). The thinner sample is very smooth while the thicker one is very rough with R_a_ value as high as 26 nm for 2 × 2 μm^2^ image.

In contrast to the thin Brush3, the thick Brush3 samples resemble Brush1 and Brush2 in that also here we observe micrometer-sized blobs; these blobs are however much larger in diameter than the features observed in Brush1 and Brush2. Moreover, the entire material appears rougher and the height differences observed in these samples reach about 200 nm.

These observations are supported by detailed surface roughness analyses (Table S1): R_a_ = 0.3 nm for Brush1, R_a_ = 0.12 nm for Brush2, and R_a_ = 0.12 nm for Brush3 (30 nm thickness), and R_a_ = 26 nm for Brush3 (300 nm).

**Figure 3:**
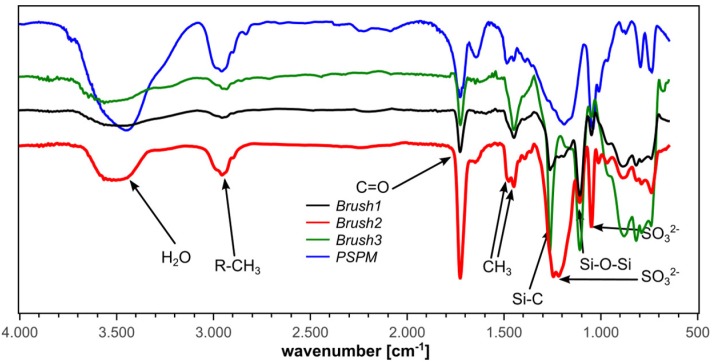
Infrared reflection absorption spectra (IRRAS) of the polymer brushes Brush1, Brush2, Brush3, and a Fourier-transform infrared (FTIR) spectrum of free (non-surface-grafted) poly(3-sulfopropyl methacrylate) potassium salt (PSPM) for comparison.

The polymer brushes were further characterized with IRRAS. Figure 3 shows representative IRRA spectra along with a Fourier-transform infrared (FTIR) spectrum of the free (i.e., not grafted to the surface) PSPM polymer made from the same monomer [15]. All characteristic IR signals of the free PSPM polymer can also be found in the spectra of the brushes. The IRRA spectra show no bands that could be assigned to the initiator moieties. This is likely due to the very low concentration of initiator groups compared to the number of monomer units in the brushes. In the case of the brushes grafted from the PEO-based initiators (Brush3) we have observed increased absorption from C–H stretching vibrations in CH_2_ groups (2933 cm^−1^) when compared to the spectra for Brush1 and Brush2 (see Figure 3). However, virtually no changes could be observed in the region 1000–1300 cm^−1^ due to strong absorption of the PSPM brush and the underlying silicon dioxide.

Besides the bands originating from the brushes, all IRRA spectra show Si–O–Si vibration bands at 1107 cm^−1^ and Si–C bands at 1263 cm^−1^. These vibrations are commonly very strong [33,34,35] and can be assigned to silicon dioxide surface layers and the silane groups in the precursors on the silicon wafer surface, respectively. Possibly, this vibration can also be assigned to a C–O–C vibration.

### 3.2. Mineralization of Polymer Brushes

As stated in the introduction, polymer brushes are interesting for (i) studying fundamental processes in (bio)mineral formation and (ii) application in biomaterials technology, in particular surface design. As a result, we have studied the ability of the polymer brushes to induce and control calcium phosphate (CP) formation. Indeed, visual inspection after mineralization shows that all samples appear optically homogeneous and are covered with a white layer of mineral.

Attempts to analyze the sample surfaces via scanning electron microscopy (SEM) or energy dispersive X-ray spectroscopy (EDXS) fail due to rapid sample charging and sample destruction. AFM fails as the samples are too rough. X-ray diffraction (XRD) only produces very noisy patterns with low count rates that cannot be analyzed further. This is consistent with earlier results on similar materials [4,5,7,8] and can be assigned to the often very low order in these materials in addition to very low sample amounts.

XPS was therefore used to determine the chemical composition (however not the crystal phase) of the mineral deposits on the surfaces (see Figure S2, Supporting Information). Especially the calcium to phosphorus (Ca/P) ratio is a useful parameter to (qualitatively) differentiate some of the CPs that may possibly be contained in the mineral films [36,37,38].

The C 1s components observed in the XPS spectra at 285.0, 286.3, and 288.9 eV are attributed to C–H, C–O, and COO groups, respectively. In combination with the corresponding O 1s peaks at 532.2 eV (O=C–O–C) and 533.5 eV (O=C–O–C) as well as the S 2p_3/2_ at 168.3 eV (SO_3_^−^ groups) these findings clearly prove the presence of the polymer brushes [39,40]. Additionally, calcium (Ca 2p_3/2_ = 347.5 eV) and phosphate (P 2p_3/2_ = 133.2 eV, O 1s = 531.1 eV) components can clearly be identified proving the successful formation of calcium phosphate within the polymer brushes [41].

Figure S2 in the supporting information shows an example of the C 1s, O 1s, Ca 2p, S 2p, and P 2p XP spectra of a Min1 surface. However, in the case of Min3 weak Si 2p peaks of Si and SiO_x_ at 99.0 and 102.7 eV, respectively, can be detected. They originate from the substrate. This corroborates the IRRAS findings and indicates a certain inhomogeneous distribution of the brushes.

All samples contain calcium, sulfur as sulfate, phosphorous as phosphate, carbon as C–H and C–O/C–N moieties along with oxygen as P–O, C=O, SiO_x_, and O=C–O compounds. In the case of Min3, also silicon as Si and SiO_x_ is detected, consistent with the IRRAS measurements described above. All samples show slight Na and Mg impurities. The Ca/P ratio determined for the entire surface is 1.92 and 1.93 for Min1 and Min3, while the ratio is 1.50 in Min2.

XPS sputter depth profiles were acquired for all mineralized samples. The rapidly increasing intensity of the bulk Si signal again indicates an inhomogeneous brush density and, therefore, it is impossible to estimate layer thicknesses. In consequence, the linear sputter time scale (rather than a nanometer scale) was retained as x-axis.

As more and more of the layer is removed and the measurement approaches the silicon substrate surface, XPS detects an increasing sulfur content (originating from the sulfonate groups in the polymer). Similarly, all samples show a high Ca, P, and O concentration at the topmost surface, which gradually decreases towards the surface of the Si wafer. The carbon signal shows relatively high C concentrations at the sample surface mainly due to adventitious carbon and then decreases in a two-step decay to below 5%, in some samples below ca. 2% towards the silicon wafer surface.

Note that XPS experiments do not detect copper signals from the polymerization reaction. This is consistent with previous data [15,18] showing that the CuCl catalyst is removed to below the detection limit of the XPS.

**Figure 4:**
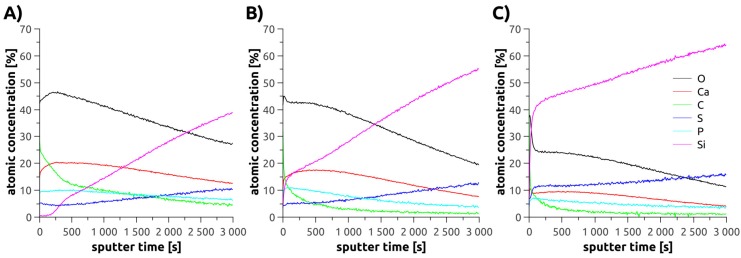
X-ray photoelectron spectroscopy (XPS) sputter depth profiles of the mineralized polymer brushes (**A**) Min1, (**B**) Min2, and (**C**) Min3. XPS raw data are summarized in the supporting information (Table S2).

**Figure 5:**
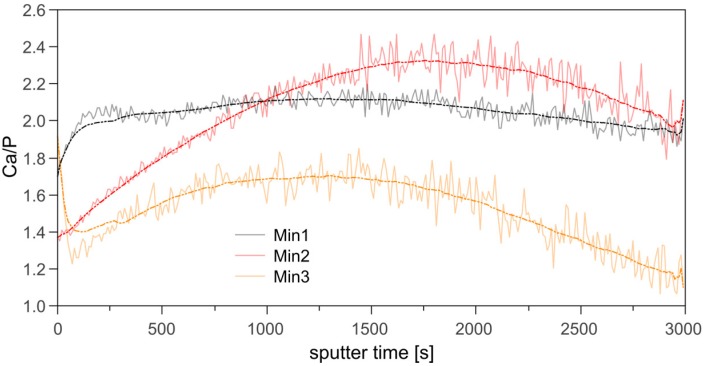
Ca/P ratios of Min1, Min2, and Min3 vs. sputter time calculated from the respective XPS sputter depth profiles in Figure 4: solid lines represent the experimental data; dash-and-dot lines are the respective sliding averages from the experimental data.

Based on the elemental composition profiles shown in Figure 4 Ca/P concentration ratios vs. sputter depth were calculated for the three samples types. Figure 5 shows that indeed there is a variation of the Ca/P ratio vs. sample depth. Moreover, there are also variations between the three types of polymer brushes. Min1 shows two regimes within the Ca/P ratio: (i) close to the surface the Ca/P ratio is around 1.7 but rapidly rises to ca. 2. After this initial increase, no further significant changes are observed until very close to the Si surface, where a small increase is observed once again.

Min2 samples are much more heterogeneous. The Ca/P ratio begins at just below 1.4 at the sample surface and increases to 2.4 before decreasing again to about 2 close to the Si surface. Very close to the Si surface, we observe the same small, but noticeable increase already described for Min1.

Min3 samples are again different from the samples described above because here the initial Ca/P ratio close to the surface is about 1.9, but then rapidly decreases to 1.4. It subsequently increases again to ca. 1.6 and then decreases to a very low value of ca. 1.2 close to the Si surface.

As shown above, XPS seems to indicate some composition variation vs. film depth. This may be assigned to the fact that the current materials are rather complex and may indeed not be entirely homogeneous throughout the entire sample depth. In principle one could use carbon or sulfur signals but XPS spectra acquired from mineralized samples also contain other elements and represent a highly complex matrix with local heterogeneities in both the films and the mineral components. Moreover, since IRRAS data (see below) suggest the formation of carbonated apatite, the carbon signal in the XPS spectra cannot solely be assigned to the polymer, which further complicates the analysis.

As stated above, XRD analysis of the samples to assign (crystal) phases is not possible. However, IRRAS analysis of the samples shows several bands that can be used for a qualitative phase assignment of the mineral phases within the polymer brushes.

**Figure 6:**
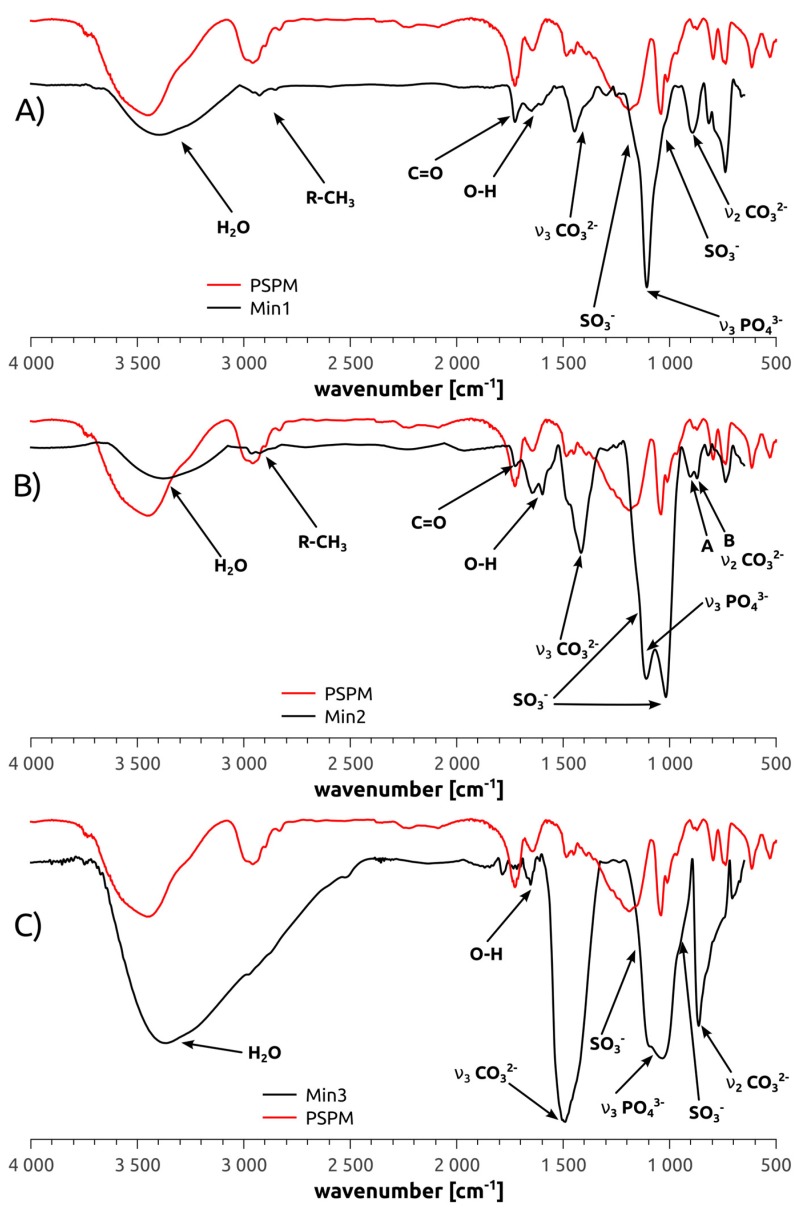
IRRAS data of the mineralized polymer brushes. (**A**) Min1; (**B**) Min2 and (**C**) Min3. PSPM refers to the neat polymer (i.e., polymer not attached to the surface, reproduced from [15] with permission, copyright American Chemical Society, 2014).

Figure 6 shows representative IRRAS data of Min1, Min2, and Min3. The broad signal observed at 1107 cm^−1^ in the spectra of Min1 is due to the ν_3_-PO_4_^3−^ vibration of apatites; possibly this signal overlaps with a Si–O–Si signal that also occurs at this position. Additional bands at 1445 and 893 cm^−1^ can be assigned to the ν_2_ and ν_3_ vibrations of CO_3_^2−^ [30,31]. Both the width of the ν_2_ and the shape of the ν_3_ vibrations indicate the presence of carbonate-substituted hydroxyapatite (CHAP) crystallized in AB-type.

IRRAS data of Min2 are similar, but show some differences. Again, the PO_4_^3−^ ν_3_ vibration is visible at 1109 cm^−1^ yet the SO_3_ and Si–O–Si at 1107 and 1263 cm^−1^ appear stronger than in Min1 and correspondingly two maxima are observed. The C=O vibration at 1726 cm^−1^ indicative of the sulfopropyl moieties of the polymer brush is more distinct than in Min1. Finally, ν_2_ vibrations caused by carbonate ions in AB-type CHAP are visible at 871 (B-type substitution) and 903 cm^−1^ (A-type substitution) of CO_3_^2−^. This observation is supported by the shape [31,42,43,44] of the ν_3_ CO_3_^2−^ band at 1416 cm^−1^. The reason for this observation may be a somewhat higher flexibility or mobility of the individual segments in Min2 or to a somewhat higher crystalline order leading to more defined vibrational modes, but this is not entirely clear at the moment.

IRRAS data of Min3 show more intense carbonate bands at 1490 and 862 cm^−1^. The ν_3_ vibration of CO_3_^2−^ is stronger than the phosphate signal at 1043 cm^−1^. A clear decision whether or not AB-type CHAP is present in Min3 can therefore not be made. This is mostly due to the fact that these bands are very broad. Shoulders on the phosphate band at ~1100 cm^−1^ can be assigned to SO^3−^ and Si–O–Si as well.

### 3.3. Cell Compatibility of Non-Mineralized and Mineralized Polymer Brushes

To assess the effect of both the plain and the mineralized brushes on cells, we have studied the behavior of AX2 *Dictyostelium discoideum* amoebae. *Dictyostelium discoideum* is a well-established model system for eukaryotic cells [45,46,47]. Three parameters were used as a measure of cell integrity after 60 h on the hybrid materials: (i) cell morphology, (ii) presence and distribution of the actin skeleton, and (iii) the integrity of the microtubules within the cells.

**Figure 7:**
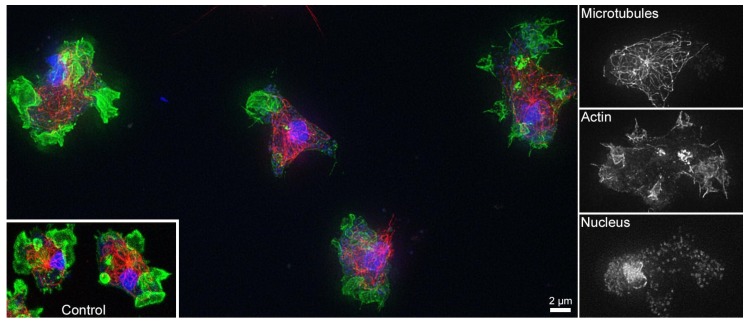
Fluorescence micrographs of *Dictyostelium discoideum* amoeba on a Min3 surface. Small images to the right show the individual red–green–blue (RGB) channels (rotated by 90°) for the cell at the upper right of the composite image showing microtubules (red), actin (green) and nuclei (blue). A representative image of control cells stained accordingly is shown in the inset. The corresponding data on Min1 and Min2 can be found in the supporting information.

Figure 7 shows representative fluorescence micrographs of *Dictyostelium discoideum* on Min3. In all cases of mineralized (Min1, Min2, and Min3) and non-mineralized (Brush1, Brush2, Brush3) brushes the *Dictyostelium discoideum* amoebae grow attached to our (hybrid) materials, showed the usual actin-rich cell protrusions and macropinocytic cups indicating normal macropinocytosis behavior in liquid medium. Moreover, they show no indications for any disruptions of the actin and the tubulin cytoskeletons compared to control cultures grown in the absence of the hybrid surfaces. This is in contrast to dying cells, which typically round up, form no macropinocytic cups and contain collapsed microtubule cytoskeletons.

## 4. Discussion

As stated in the introduction, understanding surface-controlled mineralization and being able to tailor surfaces are among the key challenges in advanced biomaterials design [9,10,11,48,49,50,51,52,53]. The current report contributes to this development and introduces a set of new surfaces that may find application in biomaterials surface development, but also enable the investigation of surface chemistry on CP deposition.

The synthesis of the polymer brushes is straightforward, but Brush3 shows relatively high surface heterogeneity likely due to non-uniform coverage of the bottom PEO layer that leads to less homogenous distribution of the initiator moieties than in Brush1 and Brush2 [54] (Figure 1, Table 1). It is also possible that the initiating moieties are buried in the PEO layer and hence initiation of the SPM polymerization is less effective which is detrimental to homogeneous polymer brush thickness) [55,56,57] than in the cases of Brush1 and Brush2.

In spite of these difference, all surfaces induce CP mineral formation suggesting useful applications for studying the above questions on mineral formation and surface design.

Moreover, as hydroxyapatite (HAP) is beneficial to both the reduction of bacterial film formation and cell colonization [58,59,60]. In addition, as polymer coatings limit bacterial growth on implants [9,10,12,13,14], the anionic brushes and the CP/polymer hybrid surfaces introduced here may potentially be useful as dual-use coating inhibiting (i) bacterial growth and (ii) promoting CP formation; this would enable the surface colonization by human cells. Indeed initial experiments using *Dictyostelium discoideum* amoebae, an established model system for eukaryotic cells [46,47], prove that both the mineral-free brushes and the mineralized surfaces do not interfere with cell growth, overall cell morphology or the appearance of the cytoskeleton, all in all parameters that usually respond very upon toxic stress factors (Figure 7, Figures S2–S7). Moreover, we have shown previously [15] that the same polymers inhibit the formation of *S. gordonii* bacteria on human dental enamel.

AFM (Figure 2) shows that Brush1 and Brush2 surfaces are quite homogeneous and IRRAS data (Figure 6) prove that all brushes are chemically identical to bulk PSPM. The characteristic IRRAS signals confirm the formation of the brushes on the surface. IRRAS and XPS (Figure 4, Figure S1) also prove that the films can be mineralized and that—although there is a slight variation in the Ca/P ratios vs. sample depth (Figure 5)—the samples are quite homogeneous in terms of their mineralization levels. Moreover the calcium phosphate deposits formed here are comparable with materials that were grown under similar conditions, including HAP, CHAP, and possibly octacalcium phosphate [37,38,61,62]. This is consistent with the Ca/P ratios found in XPS, Figure 5.

Additionally, IRRAS data also provide evidence for A-, B-, and AB substitution, mainly in the Min1 and Min2 samples, Figure 6. This is interesting because CHAP, especially AB-substituted CHAP, is enriched over senescence in human bone [32,60,63,64,65,66] and teeth [32,66,67]. The surfaces introduced here could therefore provide enhanced biocompatibility useful for implant surface modification. On a qualitative level, experiments with *Dictyostelium discoideum* amoebae support this claim, including the observation that no adverse effects of our materials on the morphology of either the actin, microtubules, or the cell nuclei is evident.

There are only a few studies on mineralized brushes for cell growth [10,68,69] but these studies are mostly based on calcium carbonate rather than calcium phosphate. Letsche et al. [10] found that mesenchymal stem cells predominantly reside in free spaces which were established within the film by photolithography.

Löbbicke et al. [5] reported the first data on poly(methacrylic acid) (PMAA) and (2-dimethyl aminoethyl) methacrylate brushes mineralized with CP. The surfaces show a high mineralization potential; this particularly applies to the PMAA brushes. Moreover, the mineralized brushes showed an enhanced cell proliferation of MC3T3 E1 pre-osteoblasts when compared to the bare non-mineralized brushes.

A similar observation was made by Van den Beucken et al. [70] who used osteoblast-like cells recovered from the bone marrow of rats. Using the layer-by-layer technique (rather than polymer brushes), these authors made coatings from DNA and poly(allylamine) or DNA and poly(d-lysine). After mineralization in 2SBF a mineral layer was observed but not characterized further. In spite of this, the mineral layer seemed to promote the delivery of osteocalcin into the extracellular matrix in cell culture, indicating that also such a coating could be interesting for biomaterial surface modification. Both the results of the current study and the other data just discussed, therefore, suggest that CP/polymer hybrid films and surfaces are a key component for the development of high-performance biomaterials surfaces. They could be particularly interesting for surfaces with a projected application in hard tissue implantology. However, in order to completely evaluate the in vitro and in vivo behavior of these surfaces, further experiments will clearly be necessary.

## 5. Conclusions

Polymer brushes based on the SPM monomer are efficient mineralization templates for the formation of CP. The minerals are a mixture of HAP, octacalciumphosphosphate (OCP), and CHAP with various substitution patterns. The current approach has several advantages over existing protocols: (i) the monomer is commercially available and reasonably cost-effective; (ii) the grafting and the polymerization process are straightforward; (iii) the mineral deposition process is simple and efficient; and (iv) viabiliy and morphology of *Dictyostelium* amoebae, a simple model for motile animal cells, were unaffected by the hybrid surfaces. These factors suggest possible applications in surface design for hard tissue implants such as bone and teeth.

## Figures and Tables

**Table 1:** Contact angle values and film thicknesses as determined using atomic force microscopy (AFM).

Identifier	Prec1	Ini1	Brush1	Prec2	Ini2	Brush2	Prec3	Ini3	Brush3
Contact angle (°)	20 ± 10	75 ± 5	7 ± 5	20 ± 10	7 5± 5	7 ± 5	15 ± 5	50 ± 5	7 ± 5
Thickness by AFM (nm)			78 ± 6			92 ± 10			30–300 ^1^

^1^ The thicknesses of Brush3 vary significantly between samples; we therefore only provide a thickness range.

## Supplementary Materials

The following are available online at http://www.mdpi.com/2073-4360/10/10/1165/s1, Table S1: Surface roughness analysis of the polymer brushes, Table S2: Chemical composition of the polymer brushes after mineralization. Figure S1: C 1s. O 1s. Ca 2p. S 2p. and P 2p XPS spectra of a Min1 surface, Figures S2–S7: Fluorescence micrographs of *Dictyostelium discoideum* amoeba on a Brush1, Brush2, Brush3, Min1, Min2 and Min3 surface, respectively. Small images to the left show the individual RGB channels (rotated by 90°) for the cell the image showing microtubules (red), actin (green) and nuclei (blue).
