# Supplementary material for: Anionic Polymer Brushes for Biomimetic Calcium Phosphate Mineralization—A Surface with Application Potential in Biomaterials
Source: Polymers (Basel). 2018 Oct 18;10(10):1165. doi: 10.3390/polym10101165 (PMC6403983; doi:10.3390/polym10101165)
Supplement: Supplementary file 1 [file polymers-10-01165-s001.pdf]

Supporting information

Anionic Polymer Brushes for Biomimetic Calcium Phosphate Mineralization–A Surface with Application Potential in Biomaterials

Tobias Mai[1], Karol Wolski[2], Agnieszka Puciul-Malinowska[2], Alexey Kopyshev[3], Ralph Gräf[4], Michael Bruns[5], Szczepan Zapotoczny[2*], Andreas Taubert[1]**

[1] Institute of Chemistry, University of Potsdam, D-14476 Golm, Germany
[2] Faculty of Chemistry, Jagiellonian University, Gronostajowa 2, 30-387 Krakow, Poland
[3] Institute of Physics and Astronomy, University of Potsdam, D-14476 Golm, Germany
[4] Institute of Biochemistry and Biology, University of Potsdam, D-14476 Golm, Germany
[5] Institute for Applied Materials and Karlsruhe Nano Micro Facility (KNMF), Karlsruhe Institute of Technology, D-76344 Eggenstein-Leopoldshafen, Germany

* **Correspondence: zapotocz@chemia.uj.edu.pl; Tel.: +48-12-686-2530**
** **Correspondence: ataubert@uni-potsdam.de; Tel.: +49-331-977-5773, Web: www.taubert-lab.net**

Table S1: Surface roughness analysis of the polymer brushes.

		Brush1	Brush2	Brush3-30nm	Brush3-300nm
Roughness average	(R_a):	0.3 nm	0.1 nm	0.1 nm	26.6 nm
Root mean square roughness	(R_q):	0.3 nm	0.1 nm	0.1 nm	32.2 nm

X-ray photoelectron spectroscopy (XPS) results

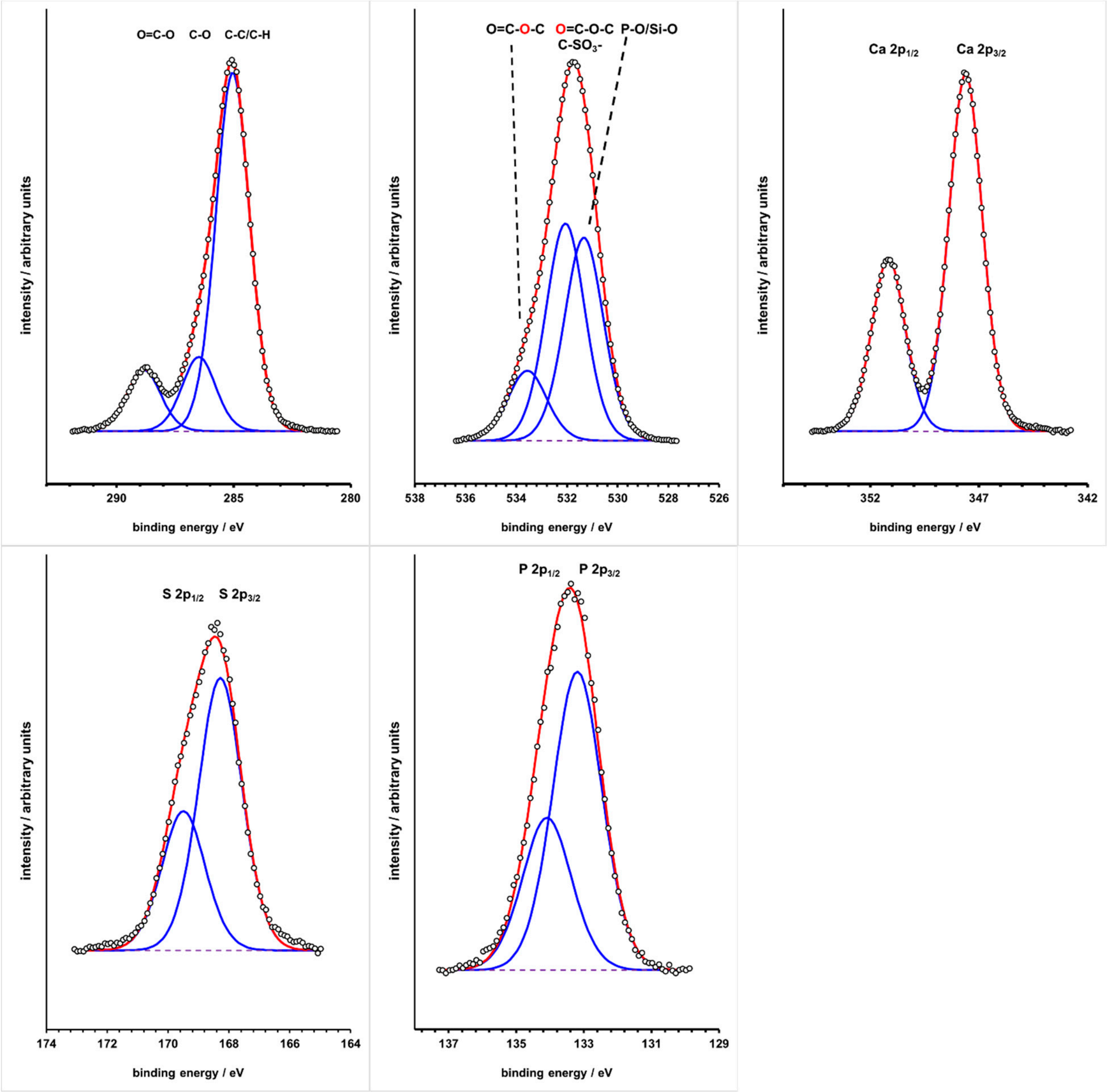

Figure S1. C 1s. O 1s. Ca 2p. S 2p. and P 2p XPS spectra of a Min1 surface

Table S2. Chemical composition of the polymer brushes after mineralization.

Sample	Si0	SiO_x	PO_4^{3-}	SO_4^{2-}	C-H	C–O / C–N	COO	Ca^{2+}	SiO_x. C=O. PO_4^{3-}		O=C–O
	Si-2p3	Si-2p	P-2p3	S-2P3	C-1s	C-1s	C-1s	Ca-2p3	O-1s	O-1s	O-1s
At%											
Min1			4.95	6.02	27.63	6.75	4.47	9.48	16.85	18.31	5.54
Min2			6.68	4.55	25.81	6.58	4.14	10.04	18.85	17.84	5.50
Min3	4.17	1.41	4.59	5.23	24.50	7.05	4.65	8.85	15.59	18.39	5.56
Peak BE [eV]											
Min1			133.3	168.4	285.1	286.4	289.0	347.7	531.3	532.3	533.6
Min2			133.4	168.5	285.2	286.5	289.0	347.7	531.3	532.3	533.6
Min3	99.2	102.9	133.2	168.5	285.2	286.5	289.0	347.7	531.3	532.3	533.6

Cell Cytotoxicity Tests

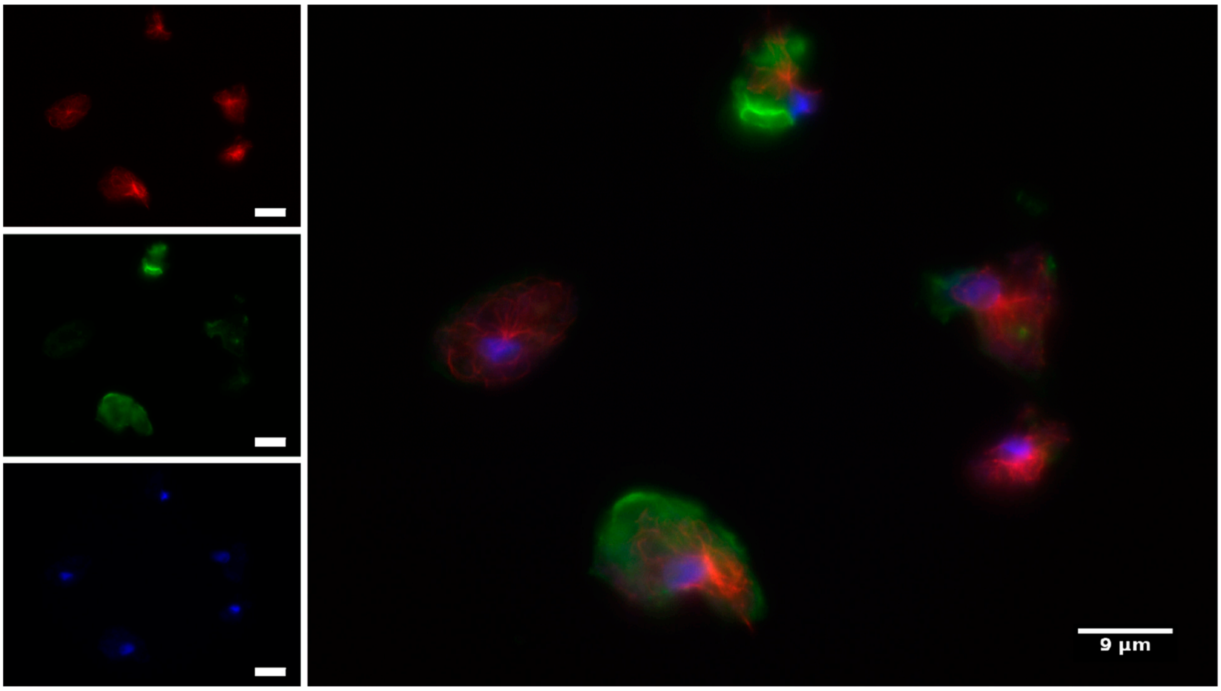

Figure S2. Fluorescence micrographs of *Dictyostelium discoideum* amoeba on a Brush1 surface. Small images to the left show the individual RGB channels for the composite image showing microtubules (red). actin (green) and nuclei (blue).

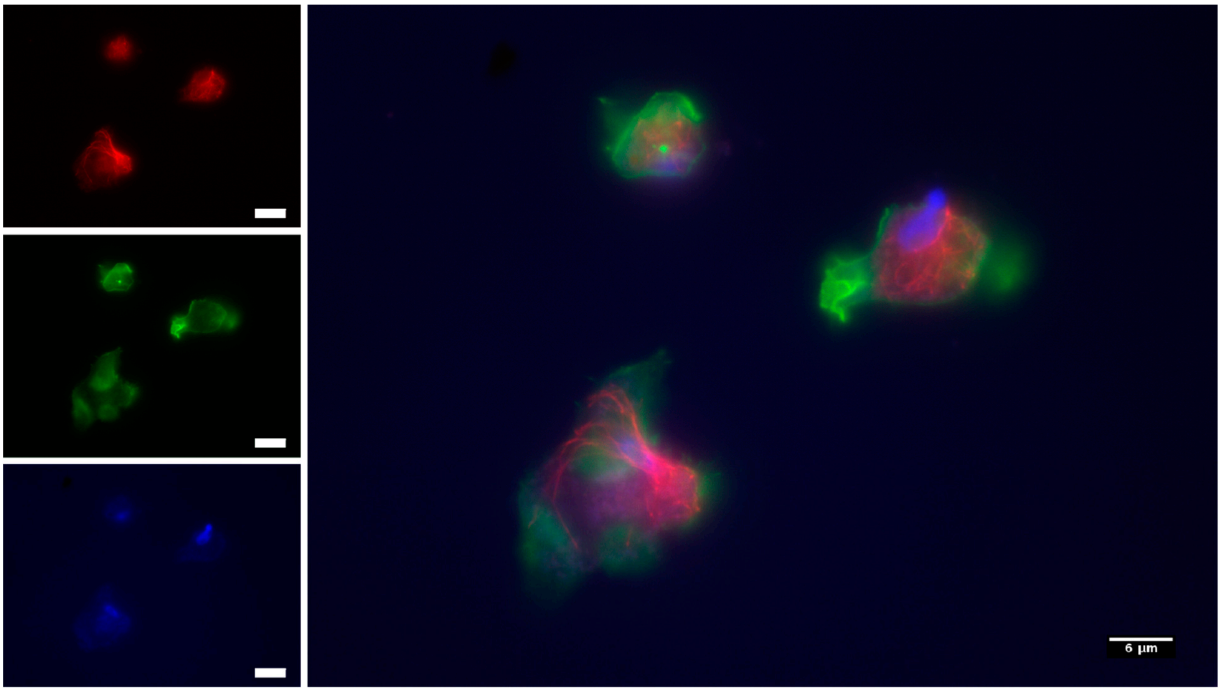

Figure S3. Fluorescence micrographs of *Dictyostelium discoideum* amoeba on a Brush2 surface. Small images to the left show the individual RGB channels the composite image showing microtubules (red), actin (green), and nuclei (blue).

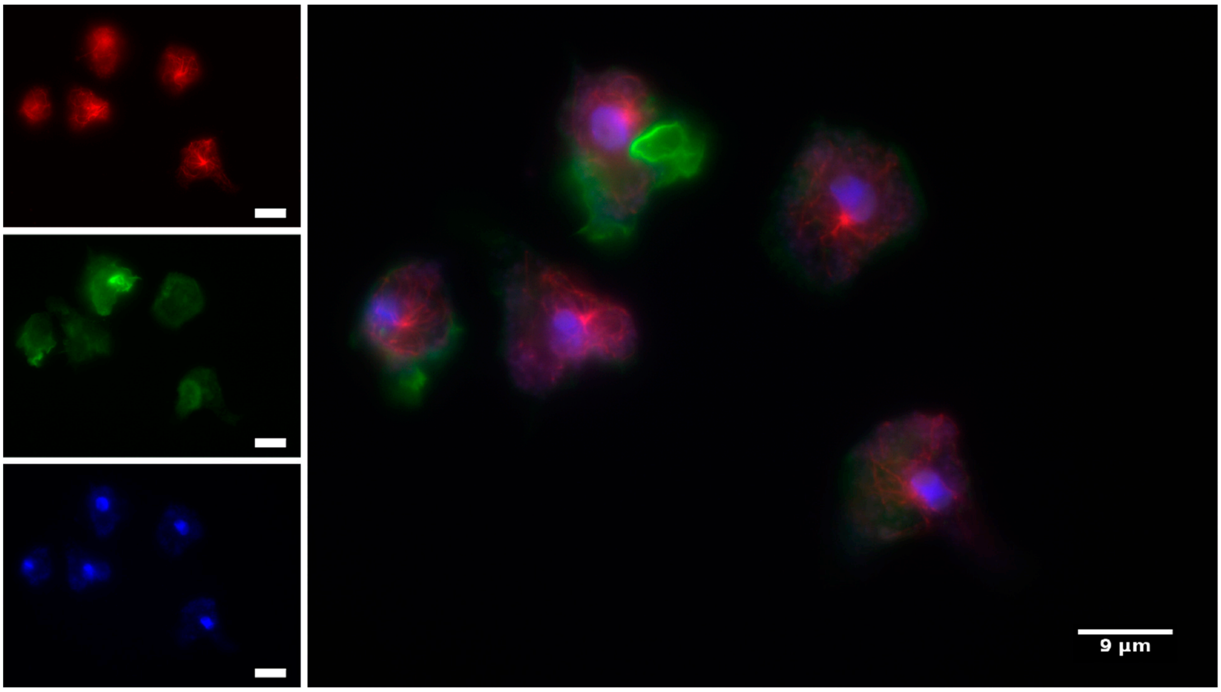

Figure S4. Fluorescence micrographs of *Dictyostelium discoideum* amoeba on a Brush3 surface. Small images to the left show the individual RGB channels for the composite image showing microtubules (red). actin (green) and nuclei (blue).

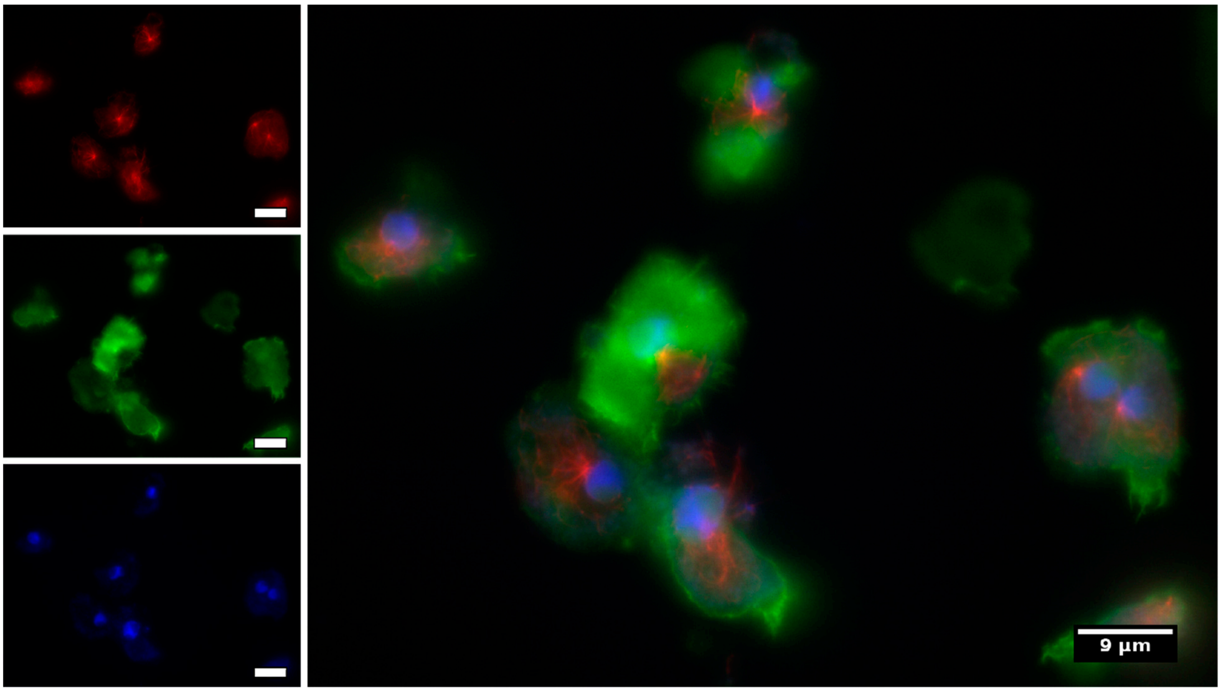

Figure S5. Fluorescence micrographs of *Dictyostelium discoideum* amoeba on a Min1 surface. Small images to the left show the individual RGB channels for the composite image showing microtubules (red). actin (green) and nuclei (blue).

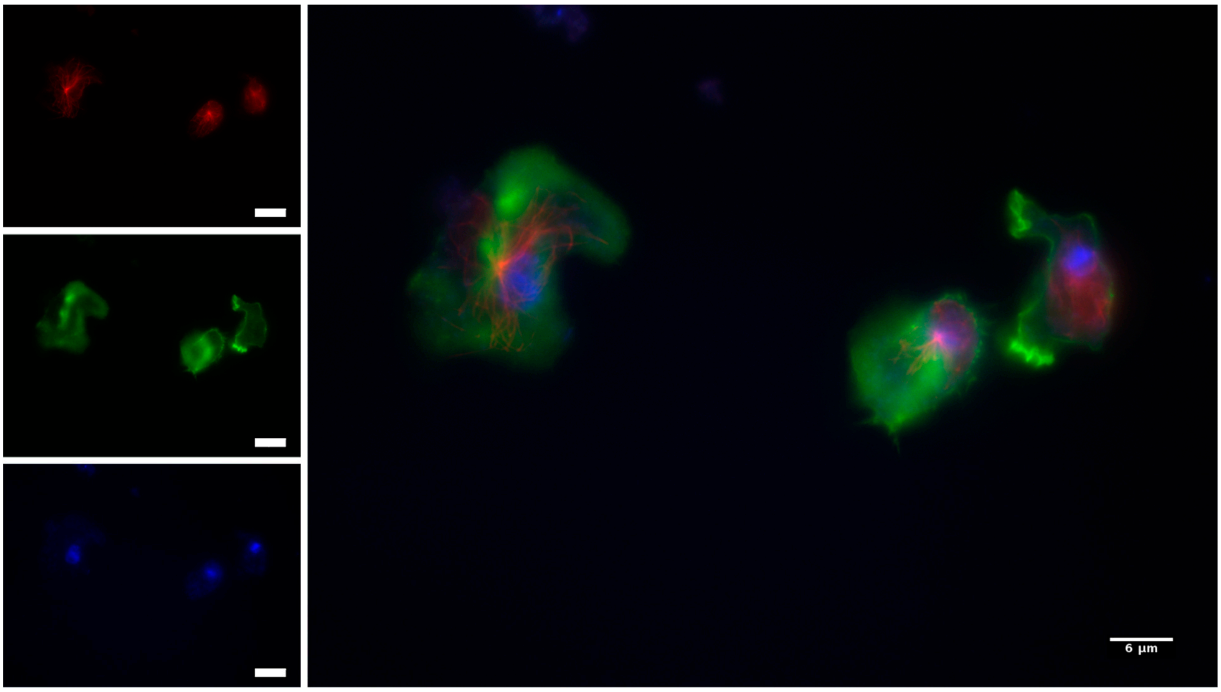

Figure S6. Fluorescence micrographs of *Dictyostelium discoideum* amoeba on a Min2 surface. Small images to the left show the individual RGB channels for the composite image showing microtubules (red). actin (green) and nuclei (blue).

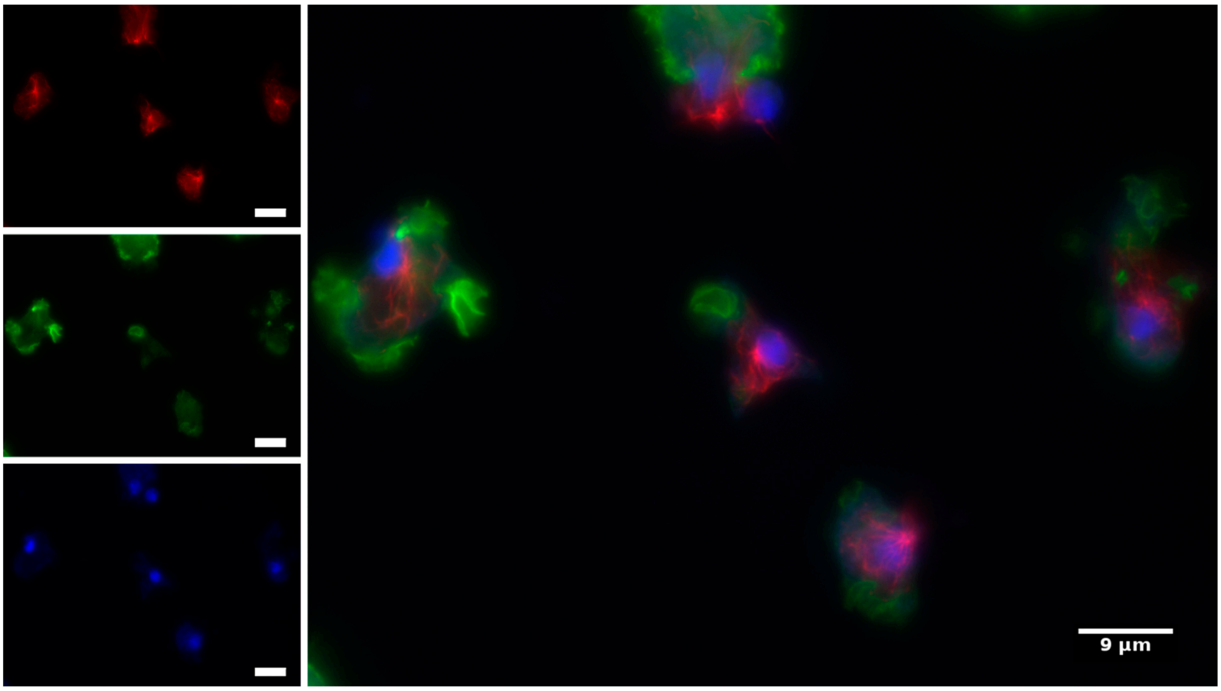

Figure S7. Fluorescence micrographs of *Dictyostelium discoideum* amoeba on a Min3 surface. Small images to the left show the individual RGB channels the upper right of the composite image showing microtubules (red), actin (green), and nuclei (blue).
